# A fistful of iron: ferritin as a vulnerability point of the brain cancers

**DOI:** 10.1038/s41419-026-08564-w

**Published:** 2026-04-03

**Authors:** Fabien Segui, Scott Kenneth Parks, Milica Vucetic, Vincent Picco

**Affiliations:** 1 Medical Biology Department, Centre Scientifique de Monaco (CSM), 98000 Monaco, Monaco; 2 Biology Department, School of Science, Technology, Engineering, and Math (STEM), Camosun College, Victoria, BC Canada

**Keywords:** CNS cancer, Cancer stem cells

## Abstract

Iron metabolism is increasingly recognized as a key player in the development and progression of various cancers. Iron is required for vital cellular processes such as energy production; however, it can also interact with reactive oxygen species to cause cellular toxicity. Consequently, a host of proteins coordinate iron homeostasis, and ferritin stands out as a promising therapeutic target due to its pivotal role in buffering cellular iron levels. This review explores the relevance of ferritin in brain cancers, shedding light on how it influences the biology of both tumor cells and cancer stem cells (CSCs), a population of tumor cells that is notable in their resistance to conventional treatment strategies. Ferritin plays a critical role in protecting against oxidative stress and boosting resistance to ferroptosis, a form of cell death often evaded by CSCs. Development of cutting-edge strategies designed to target ferritin, including ferritinophagy-inducing compounds and novel redox-based therapies that can capitalize on the iron dependency of CSCs is discussed in context. We propose that the iron addiction of brain cancer cells provides a specific susceptibility, whereby removing their iron buffering mechanism via targeting of ferritin can result in favorable treatment outcomes, including the induction of iron-dependent cell death. Future studies on the modulation of ferritin offer a ground-breaking therapeutic strategy to undermine CSC-driven tumor growth, overcome resistance to conventional therapies, and ultimately improve treatment outcomes for patients battling brain cancers.

## Key facts

- Brain cancer cells (including cancer stem cells (CSCs)) from glioblastoma (GBM) and medulloblastoma (MB), rely on high iron uptake and storage to support proliferation, survival, and stemness and thus exhibit an “iron addiction”.
- Ferritin is the primary intracellular iron storage and buffering system, protecting brain CSCs from iron-induced oxidative stress and ferroptosis.
- Elevated ferritin expression in brain tumor cells correlates with higher tumor grade, CSC maintenance, resistance to therapeutics, and worse patient prognosis, highlighting its role in tumor aggressiveness.
- Targeting ferritin via ferritinophagy inducers (e.g., NCOA4 modulation, MMRi compounds, high-dose vitamin C) can elevate labile iron, generate ROS, and sensitize brain cancer cells—particularly CSCs—to ferroptosis or other iron-dependent cell death pathways.
- Modulation of ferritin may enhance the efficacy of conventional therapies (radiotherapy, chemotherapy) by increasing ROS-mediated cytotoxicity, overcoming CSC-driven resistance, and reducing tumor relapse.

## Open questions

- How can ferritin-targeted therapies selectively induce ferroptosis in brain CSCs without causing neurotoxicity in normal neurons or glial cells?
- What are the precise molecular mechanisms by which ferritin levels regulate CSC stemness, proliferation, and survival in brain tumors?
- How do different subtypes of brain CSCs (e.g., embryonic-like vs. epithelial-like origins) respond to ferritin modulation, and how does this affect therapy outcomes?
- Which strategies can improve blood–brain barrier (BBB) penetration and CNS bioavailability for ferritin-targeting compounds while maintaining tumor specificity?
- Can ferritin expression or labile iron levels serve as predictive biomarkers for patient stratification and optimization of ferroptosis-based therapies in brain cancer?

## Introduction

**Fig. 1:**
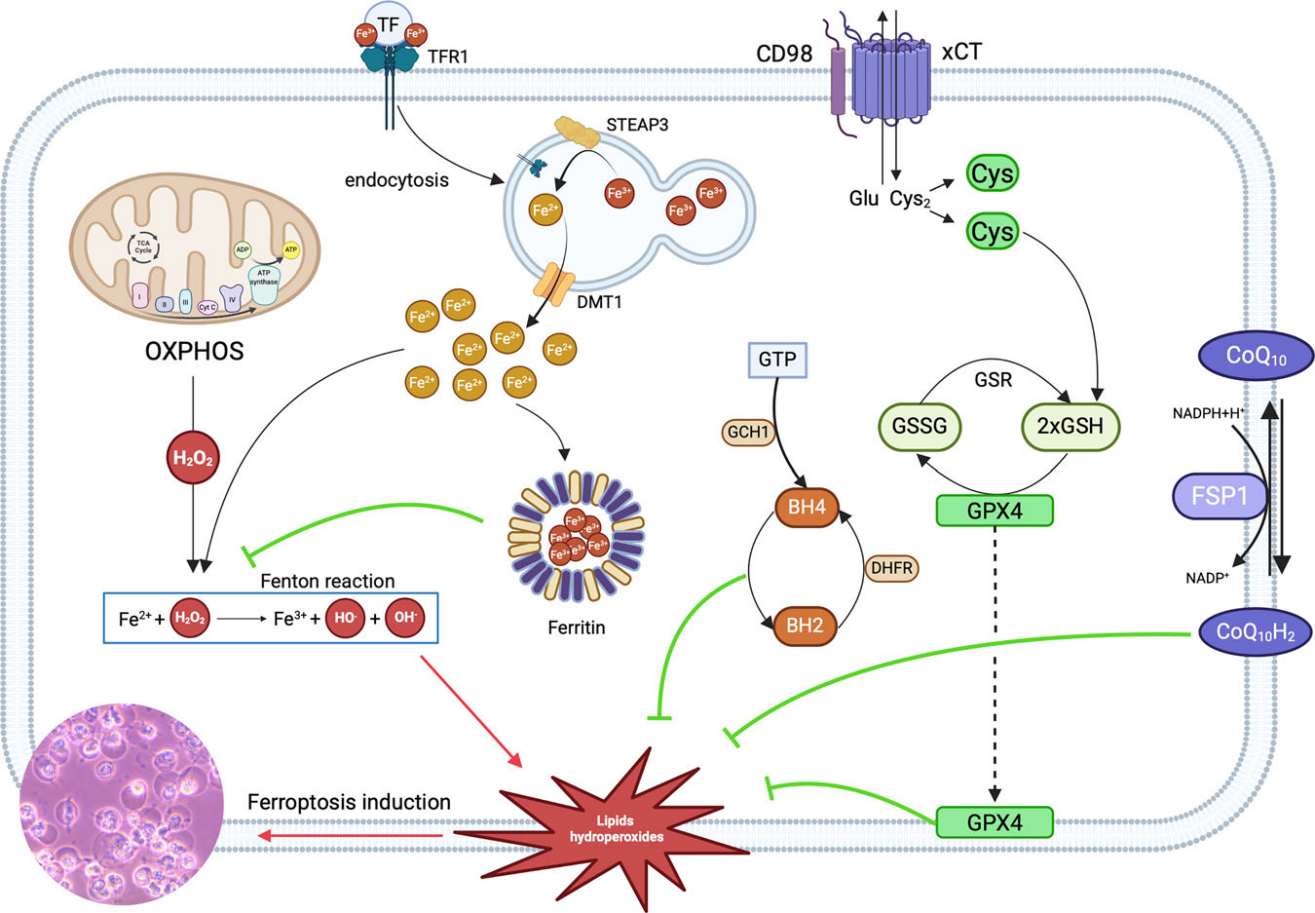
The central role of ferritin in ferroptotic cell death (explained in the text). Ferroptosis is an iron-dependent form of cell death characterized by the accumulation of lipid hydroperoxides in cell membranes. In the cell, iron exists as either a free active ferrous ion (Fe2+) or stored as a ferric ion (Fe^3+^). Fe^2+^ ions interact with reactive oxygen species (ROS), such as hydrogen peroxide (H2O2), or lipid hydroperoxides (LOOH), in the process known as the Fenton reaction. This interaction gives rise to lipid hydroperoxyl radicals (LOO•) and if these propagate throughout the membrane compartment, it leads to the loss of membrane integrity, which inevitably results in cell swelling, membrane rupture and death. Given that ferroptosis is a form of non-programmed cell death, it poses significant risks to cells if it is not meticulously regulated. To reduce this phenomenon, antioxidant pathways can remove lipid hydroperoxides by reducing them to non-toxic lipid alcohols. One of the key antioxidant pathways involves the solute carrier family 7 member 11 (SLC7A11, also known as xCT). This transporter facilitates the entry of cystine into the cell in exchange for glutamate. Once inside, cystine is reduced to cysteine, which serves as a precursor for the synthesis of glutathione (GSH). GSH is a crucial cofactor for glutathione peroxidase 4 (GPx4), an enzyme that prevents the formation of lipid radicals by converting lipid hydroperoxides into non-toxic lipid alcohols, thereby limiting the propagation of lipid hydroperoxides and reducing ferroptosis. There are also glutathione-independent pathways, such as the ferroptosis suppressor protein 1 (FSP1), formerly known as apoptosis-inducing factor mitochondrial 2 (AIFM2). FSP1 uses NADP + /NADPH to reduce coenzyme Q10 (CoQ10) or vitamin K to their respective hydroquinone, CoQ10H2 and vitamin K hydroquinone. These compounds act to neutralize free radicals, thereby reducing lipid hydroperoxides in membranes. Another significant pathway involves GTP cyclohydrolase 1 (GCH1), which generates tetrahydrobiopterin (BH4), a reducing agent that helps remodel lipids and prevent PUFA depletion, thereby inhibiting ferroptosis. In contrast, ferritin can act as a buffering mechanism to store free iron and thus reduce the amount of reactive oxygen species generated by the Fenton reaction. BH2 dihydrobiopterin, CD98 cluster of fifferentiation 98, Cys cysteine, CYT C cytochrome c, DHFR dihydrofolate reductase, Glu glutamate, GSR glutathione reductase, GSSG oxidized glutathione (glutathione disulfide), GTP guanosine triphosphate, NADP⁺ nicotinamide adenine dinucleotide phosphate (oxidized form), NADPH nicotinamide adenine dinucleotide phosphate (reduced form), OXPHOS oxidative phosphorylation, PUFA polyunsaturated fatty acid, STEAP3 six-transmembrane epithelial antigen of prostate 3, TF transferrin, TFR1 transferrin receptor 1. *Created with BioRender.com*.

Cancer cells require increased iron availability compared to normal cells, underscoring the critical importance of modulating iron homeostasis to survive [1]. Furthermore, the population of cancer stem cells (CSCs) within any tumor (now widely accepted to be the major source of resistance to conventional therapies), exhibits more pronounced iron dependency based on import and storage capacities compared to non-stem cancer cells [2, 3]. Brain cancer cells have been shown to exhibit an “iron addiction” to support their proliferation, survival, and maintenance of the CSC pool [2, 3]. This suggests that targeting iron metabolism could be a promising strategy to exploit the vulnerabilities of cancer cells, thus improving treatment efficacy and mitigating resistance. Combined with this ‘iron addiction’ in the brain and other cancers, the past two decades have experienced a burgeoning interest in the study of ferroptosis as an anti-cancer strategy. Ferroptosis was first defined [4] as a distinct form of regulated cell death induced by erastin. Erastin blocks cystine uptake, depleting glutathione and inactivating glutathione peroxidase 4 (GPX4), the enzyme responsible for detoxifying phospholipid hydroperoxides. Iron was shown to drive ferroptosis through Fenton chemistry [5], where ferrous iron (Fe^2+^) reacts with hydrogen peroxide (H_2_O_2_) to generate hydroxyl radicals (HO^•^) that initiate and amplify lipid peroxidation (Fig. 1). Ferroptosis is thus recognized as an iron-dependent, lipid peroxidation-driven form of cell death, mechanistically distinct from apoptosis or necroptosis. It can be prevented by GPX4-mediated detoxification of lipid peroxides [6–9] or by limiting cellular iron availability [10–14] (Fig. 1). However, the sequestration of iron by ferritin complexes, which play a pivotal role in minimizing redox reactions between iron and reactive oxygen species (ROS) to reduce ferroptosis events, has received less attention in the literature. In this review, we expand on ferritin complexes being the source of cellular iron ‘buffering capacity’ and thus, how they provide a therapeutic vulnerability point for cancer cells that are metabolically addicted to iron. Numerous anti-cancer studies have proposed targeting iron with chelators to decrease the labile iron pool (LIP), however, the non-specificity of this strategy results in serious side effects [15]. Therefore, it is imperative to find alternative methods of iron manipulation in a cancer-specific manner. As we will illustrate, this may be desirable for iron depletion to reduce tumor cell growth but also to achieve sudden iron elevation to promote ferroptosis and/or other iron-dependent cell death pathways. We propose that targeting ferritin, due to its crucial role in maintaining iron homeostasis within cancer cells, represents a viable target in this context.

## The role of iron in brain (patho)physiology

Biological availability of iron is often restricted due to its tendency to form insoluble compounds and therefore, systemic absorption, cellular distribution and storage of iron are crucial [16]. Under normal conditions, consistent iron availability is achieved via recycling of the heme iron in red blood cells at the end of their lifespan, combined with dietary iron absorption in the small intestine. Iron then circulates in the bloodstream, while surplus iron is stored in the liver as ferritin, which can be utilized when the demand arises.

Iron is indispensable for cellular metabolism and energy production in the brain as a core component of cytochromes [17] and iron–sulfur clusters [18], which are fundamental to the electron transport chain in mitochondria [19]. A deficiency in iron can impair mitochondrial oxidative phosphorylation, reducing ATP production and compromising neuronal performance [20, 21]. Excess free iron can catalyze ROS production, leading to oxidative stress and neurodegenerative diseases. For example, in Alzheimer’s disease, increased free iron levels are found in amyloid plaques and neurofibrillary tangles, contributing to oxidative stress and neuronal injury [22, 23]. In Parkinson’s disease, excess labile iron in the *substantia nigra* promotes the aggregation of the disease hallmark alpha-synuclein [24, 25]. Similarly, iron and ferritin aggregates in CNS cells affect movement and cognition, a characteristic of neuroferritinopathy [26]. Conversely, consideration of iron deprivation is crucial during brain development, where adequate iron levels are critical for proper myelination, neuronal differentiation/maturation [27–29], and neuronal stem maintenance/proliferation [30, 31]. Iron deficiency during crucial developmental periods can lead to long-term cognitive challenges and motor deficits [32, 33]. Furthermore, in multiple sclerosis, low iron availability is associated with demyelination and neuroinflammation [34].

### Iron homeostasis in the CNS-ferritin and cancer vulnerability

**Fig. 2:**
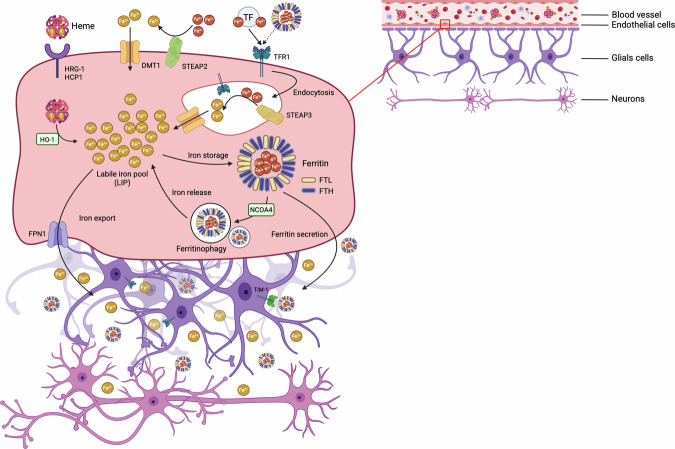
Mechanisms of iron import, storage, and export in endothelial cells of the blood–brain barrier (BBB). Transferrin (TF)-bound iron is internalized via endocytosis upon binding to the transferrin receptor 1 (TFR1). Within the endosome, ferric iron (Fe^3+^) is reduced to ferrous iron (Fe^2+^) by the six-transmembrane epithelial antigen of the prostate 3 (STEAP3) and subsequently exported into the cytoplasm by divalent metal transporter 1 (DMT1). Heme oxygenase 1 (HO-1) can also release iron from heme, which is imported via the HRG-1/HCP1 transporters. DMT1 is also involved in transporting Fe^2+^ directly into the cell. Fe^2+^ is previously reduced from Fe^3+^ by STEAP2 at the plasma membrane. Once in the cytoplasmic labile iron pool (LIP), iron can be used for cellular needs, stored in ferritin complexes or exported via ferroportin-1 (FPN1). Under iron-restricted conditions, the nuclear receptor coactivator 4 (NCOA4) protein binds to the ferritin complex, facilitating its lysosomal degradation (ferritinophagy) and enabling the release of previously stored iron. Endothelial cells meet the iron requirements of glial and neuronal cells either through the export of free iron by FPN1 or via ferritin-bound iron stores. For instance, T-cell immunoglobulin and mucin domain-containing protein-1 (Tim-1), expressed on oligodendrocytes, binds ferritin and facilitates its internalization. STEAP2 six-transmembrane epithelial antigen of the prostate 2, FTH ferritin heavy chain, FTL ferritin light chain. *Created with BioRender.com*.

The blood–brain barrier (BBB) serves as the regulatory entry point for iron into the brain and governs its bioavailability (explained in Fig. 2). Following iron transfer across the BBB, free iron (or ferritin) is then metabolized by CNS cells, a pathway also available for brain cancers. The ferritin complex is the only known protein complex that can act as both an iron donor and play a role in buffering capacity within the cells to balance possible iron toxicity. Overall, ferritin is thus essential for both neurogenesis and neuronal cell survival, due to its vital role in maintaining iron homeostasis. Ferritin consists of two subunits: the light chain (L-ferritin or FTL) and the heavy chain (H-ferritin or FTH). Cytoplasmic ferritin sequesters iron into a biochemically inert form as a result of the ferroxidase activity of the H subunit [35], with each ferritin complex storing up to 4500 iron atoms as ferric ions (Fe³⁺) [36]. Therefore, cells can have a large total quantity of iron sequestered in ferritin complexes that is not biologically active.

Ferritin, therefore, acts as an iron buffer allowing cells to resist increased oxidative pressure. This property is exploited by cancer cells, which develop a strong dependence on iron to satisfy the heightened metabolic demands associated with their uncontrolled proliferation and invasive behavior. This function appears particularly crucial in brain tumors, which exhibit a pronounced dependency on iron. These insights from CNS pathophysiology illuminate that disruption of iron levels, by removing their buffering capacity via targeting of ferritin, could provide a beneficial role in sensitizing brain cancers to iron-dependent cell death and reduced proliferation, as we will explore in this review.

## The role of iron in cancer

### Cancer initiation

Iron plays a critical role in cancer initiation due to its ability to catalyze the formation of ROS, which induces oxidative damage to cellular DNA, resulting in mutations, genomic instability and promotion of malignant cell transformation [37, 38]. Additionally, iron overload can enhance cellular proliferation and sustain chronic inflammatory responses, both of which are key processes in tumorigenesis [39]. These studies highlight the necessity for tight regulation of iron metabolism to prevent iron-induced DNA damage and cancer initiation. Furthermore, a large-scale cohort study found that higher intake of heme iron is significantly associated with an increased overall cancer risk (reviewed in [40]). In line with this, iron overload, either by parenteral administration of iron dextran or by iron-rich food, has been shown to increase the risk of developing cancer in rodents, rabbits, and humans [41–45].

### Cancer progression

**Fig. 3:**
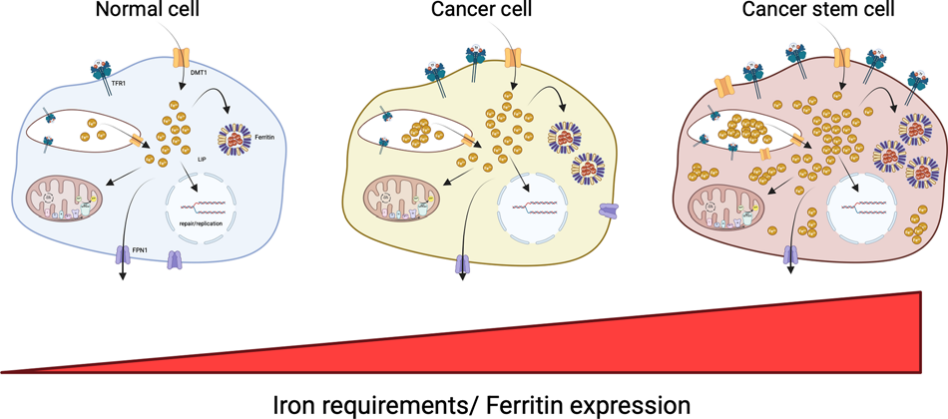
Overview of differential expression patterns of cellular iron regulators that achieve varying levels of iron homeostasis when comparing normal cells to cancer cells and cancer stem cells (CSCs). Cancer cells harbor higher iron requirements than normal cells and meet these demands via increasing iron import and storage through upregulation of TFR1, DMT1 and ferritin in addition to decreasing iron export through downregulation of FPN1. These observations are even more pronounced in CSCs in comparison to cancer cells. See Fig. 1 for a reminder of abbreviations, iron-regulating proteins, and concepts illustrated in these three cell types. TFR1 transferrin receptor 1, DMT1 divalent metal transporter 1, FPN1 ferroportin-1. *Created with BioRender.com*.

Furthermore, cancer patients frequently demonstrate elevated serum ferritin levels when compared to healthy individuals [46]. This observation has been reported in a wide range of cancers, including breast [47], lung [48], pancreas [49], colorectal [50] and oral [51]. Furthermore, studies have shown that high serum ferritin levels are a marker of poor prognosis in patients with pancreatic [52], neuroblastoma [53], prostate [54], colorectal [55] and liver [56] cancers. Additionally, ferritin levels have been suggested as a marker for post-treatment prognosis, particularly in pancreatic cancer [57]. Although the ultimate reason for high circulating levels of ferritin in these cases may be challenging to identify, elevated serum ferritin levels provide access to adequate iron quantities for supplying tumors that possess enhanced iron metabolism. Increased iron metabolism in cancer cells is orchestrated by upregulation of iron import via TFR1 [58–61] and DMT1 [62], and/or downregulation of iron export via FPN1 [60] (Fig. 3). This combination enables cancer cells to accumulate substantial amounts of iron, which supports their high metabolic demands to promote growth, survival and invasion/metastasis [62–64]. This reveals part of the role that iron can play as a risk factor in cancer development. In accordance with this, studies where iron accumulation was forced in tumor cells, either by overexpression of TFR1 or through iron-rich nutrition, demonstrated enhanced tumor growth [65, 66]. Conversely, iron depletion achieved through either dietary restriction [67], iron chelation [68], blocking iron import [69] or increased iron export [70], have been shown to limit tumor progression.

In established tumors, we can observe the influence of iron on the mechanistic target of rapamycin (mTOR) [71]. Cancer cells enhance mTORC1 activity, which promotes gene transcription and protein synthesis to control cell proliferation, metabolism, and autophagy [72–74]. Reduction of cellular iron via chelators, including ciclopirox (CPX), di-2-pyridylketone 4,4-dimethyl-3-thiosemicarbazone (Dp44mT), and deferoxamine (DFO) [75], in addition to TFR1 silencing [64] have been shown to inhibit mTORC1 activity in cancer cells. mTORC1 is well known to be controlled by variations in levels of ATP, amino acids, oxygen, DNA damage and oxidative stress [74]. These additional regulatory implications for iron homeostasis in mTORC1 activity highlight the close relationship to this key signaling pathway in cancer cells.

### Cancer invasion and metastasis

A growing body of literature suggests that iron influences the epithelial–mesenchymal transition (EMT), a key process in cancer cell invasion and metastasis. Namely, an increase in heme oxygenase 1 (HO-1) activity in breast cancer cells leads to a transient release of free iron and a consequent increase in ROS content, promoting the expression of matrix metalloproteinase 1 and cell migration [76]. In the pro-inflammatory environment, iron overload enhanced breast cancer cell migratory capacity via the IL-6/JAK2/STAT3 signaling pathways [77]. The excess iron-induced increase of mesenchymal and metastatic biomarkers in hepatocellular carcinoma cells appears to be, at least in part, due to alterations in the expression of EMT transcription factors SNAI1 and SNAI2 [78]. Furthermore, chronic exposure to excess iron leads to heme-mediated export of the tumor suppressor p53 out of the nucleus in both in vitro and in vivo models of pancreatic cancer, resulting in the promotion of EMT [79]. In addition, studies utilizing iron chelators have proven to inhibit EMT. One of the most convincing cases in this regard is the upregulation of N-myc downstream-regulated gene 1 (NDRG1) by iron chelators [80, 81]. NDRG1, often referred to as a metastasis suppressor protein, has been shown to be a potent inhibitor of transforming growth factor beta (TGF-β)-induced EMT in colon and prostate cancer [82]. Combined, these data suggest an important role for iron in EMT and ultimately cancer invasiveness. This provides further justification to examine ferritin’s role in iron buffering for not only the control of tumor cell proliferation, but also in the context of controlling dissemination.

### Cancer adaptation and tumor environment

The tumor microenvironment (TME) is composed of stromal, immune, vascular, and molecular components that interact closely with cancer cells to influence their survival, proliferation, and invasive capacity. Within the TME, tumor-associated macrophages (TAMs) often acquire a tumor-supportive phenotype, promoting angiogenesis and enabling immune evasion. Intracellular iron accumulation has been shown to drive macrophage polarization toward the M2 phenotype, which is associated with the secretion of anti-inflammatory cytokines, promotion of neovascularization, and suppression of anti-tumor immune responses [83]. In a glioma model, high expression of the ferritin light chain subunit (FTL) in TAMs was shown to promote M2 polarization, thereby contributing to tumor progression [84]. Conversely, iron deprivation favors polarization toward the M1 phenotype, characterized by enhanced production of pro-inflammatory cytokines and increased capacity to phagocytose and kill tumor cells [83]. Thus, the regulation of iron metabolism in TAMs represents a critical lever for modulating their function within the TME, with direct implications for tumor progression.

Cancer-associated fibroblasts (CAFs) have also been identified as active contributors to tumor progression in several solid malignancies [85] and have been shown to promote tumor growth by modulating iron metabolism [86]. In prostate cancer, a subset of iron-loaded CAFs, referred to as FerroCAFs, utilize intracellular iron to activate KDM6B, an iron-dependent epigenetic regulator. This activation facilitates chromatin remodeling and enhances the transcription of genes encoding myeloid cell-associated proteins. Consequently, FerroCAFs recruit immunosuppressive myeloid cells that thereby attenuate the antitumor immune response [86]. Hypoxia is a hallmark of the TME where it alters cellular functions, many of which relate to the metabolism of tumor, stroma and infiltrating immune cells. The well-described transcription factor, HIF-1α, that is stabilized under hypoxic conditions, drives gene expression pattern changes via binding to hypoxia-responsive elements (HRE) in the genome [87]. Critical iron-regulatory genes that are upregulated by HIF-1α, including FTH, TFR1 and DMT1, highlight the intrinsic relationship between iron metabolism and hypoxia as a fundamental aspect of cancer cell adaptation within the tumor microenvironment [88]. This regulatory mechanism ensures that cancer cells are capable of maintaining iron homeostasis and their proliferative capacity despite oxygen deprivation. Furthermore, iron levels directly influence HIF-1α stability, as prolyl hydroxylases (PHDs) responsible for HIF-1α degradation require Fe²⁺ as a cofactor. Under conditions of iron depletion or chelation, PHD activity is impaired, resulting in HIF-1α stabilization even in normoxia [89]. Thus, iron availability not only responds to hypoxia but also actively regulates the hypoxic response, highlighting the bidirectional link between iron metabolism and HIF signaling. Notably, HIF-1α stabilization under iron-depleted conditions increases VEGF production and promotes angiogenesis, thereby supporting cancer survival and progression, as demonstrated in breast cancer models [90]. This interplay between iron metabolism and hypoxia is particularly significant in the context of CSCs, which reside in hypoxic niches [91] and leverage these adaptive responses to sustain their stemness properties and resistance to therapeutics (reviewed [92]).

Overall, iron profoundly shapes multiple stages of cancer development, from initiation and progression to metastasis and tumor microenvironment adaptation by supporting proliferation, survival, and immune evasion. These same iron-dependent mechanisms are increasingly recognized in CSC. Thus, understanding how iron metabolism sustains CSC maintenance and function represents a key step in elucidating iron’s full impact on cancer biology.

## Iron and CSCs

CSCs are widely recognized as the main contributors to both therapeutic resistance and relapse. CSCs are a subpopulation of cells within the TME with the ability to self-renew and differentiate, thereby contributing to cancer progression, metastasis, and resistance to conventional therapies (reviewed in ref. [93]). CSCs have distinct metabolic requirements compared to non-CSCs (and normal cells), with a heightened dependency on iron (reviewed extensively in [94]) (Fig. 3). By tightly controlling iron import, storage and export, CSCs can avoid iron-induced oxidative stress and maintain their viability. In this context, targeting ferritin could be particularly valuable for eliminating CSCs in addition to other cancer cells, potentially enhancing treatment efficacy and reducing the risk of relapse.

### High iron demands in CSCs

CSCs upregulate iron import through classical pathways such as TFR1/DMT1 (as shown in Fig. 3) [3, 95, 96]. Alternatively, CD44, a well-known stem cell marker [97], has been implicated in a hyaluronate-dependent iron endocytosis [98]. Another well-known stem marker, CD133, has also been characterized to support iron uptake by the TF/TFR1 pathway [99], suggesting that enhancement of iron uptake in CSCs is necessary to meet their increased iron requirements. Additionally, iron retention in CSCs can be achieved by repressing FPN1 and thus preventing export of cellular iron. For example, cholangiocarcinoma stem-like cells have been shown to retain iron due to low FPN1 expression levels and elevated ferritin content [100]. In ovarian cancer, tumor-initiating cells (possessing CSC properties) show a reduction in FPN1 and an increase in TFR1 when transduced with the c-MYC oncogene [100]. CSCs in other cancer types also increase their iron storage capacity by upregulating ferritin expression [3, 101] or ferritin uptake via L-Ferritin receptor Scavenger Receptor Class A member 5 (SCARA5) [102].

### Iron levels and expression of stemness markers

Conversely, elevated iron levels also appear to drive stem cell identity by increasing expression of CSC markers. For instance, in lung cancer, Müller and colleagues [98] demonstrated that iron accumulation promotes CSC proliferation, spheroid formation, and the expression of stem cell markers, such as SOX9, potentially via a ROS-dependent mechanism. Transcriptional regulation via the action of the α-ketoglutarate (KG)-dependent demethylase PHF8, which induces demethylation of histone H3 lysine 9 dimethyl (H3K9me2), has been shown to regulate CD44 expression in an iron-dependent fashion [98]. Combined, these studies show the presence of a feedback loop in which CSCs are capable of elevating intracellular iron levels, which strengthens the stem-like characteristics of the cells within the tumor mass. Supporting data have further shown that high TFR1 expression results in high iron levels in hepatocellular carcinoma CSC, increasing their malignant behavior with a potential role in stem maintenance [103].

### Intracellular iron maintains the CSC profile

Epigenetic modifications, including DNA methylation, histone modification, and chromatin remodeling are a major hallmark of CSCs. Among the key regulators of these epigenetic modifications are metabolites from the tricarboxylic acid (TCA) cycle. Namely, α-KG is an essential substrate for the α-KG-dependent dioxygenases (α-KDDs), which include the subfamilies Jumonji C-domain lysine demethylases (JmjC-KDMs) and ten-eleven translocation (TET) DNA cytosine-oxidizing enzymes [104]. Many cancers, including gliomas [105] are characterized by decreased α-KDD activity. This reduction is often caused by oncogenic mutations in metabolic enzymes like succinate dehydrogenase (SDH), fumarase (FH), and isocitrate dehydrogenase (IDH1/2), leading to the accumulation of metabolites such as fumarate, succinate, and 2-hydroxyglutarate (2HG), respectively (reviewed in refs. [104, 106]). The accumulation of these metabolites has been shown to inhibit α-KDD activity (most likely due to α-KG depletion), thereby promoting stem cell differentiation [107, 108]. Besides α-KG, iron (Fe²⁺) serves as another essential cofactor for the activity of the α-KDD enzymes. Consequently, increasing the Fe²⁺ pool could promote α-KDD activity and increase stemness within the tumor. Zhang and colleagues showed that the catalytic activity of JmjC domain-containing histone demethylase 1 (JHDM1) is halted when ascorbate is withdrawn from the in vitro assay, most likely due to the reduced content of catalytically active Fe^2+^ (reviewed in ref. [109]). This further reinforces the role of cellular iron for maintenance of the CSC pool and will be discussed in the context of ferritin as a therapeutic target for this cell population.

## Ferritin expression in brain cancer

### Serum ferritin

A cohort of pediatric cancer patients revealed that elevated serum ferritin levels were significantly associated with an increased incidence of treatment-related complications [110]. Elevated ferritin levels in serum and cerebrospinal fluid have also been demonstrated in glioblastoma (GBM) patients [111, 112], while data for patients with medulloblastoma (MB) or other gliomas are currently lacking. However, it is likely that the high serum ferritin observed in other cancers may be a generic cancer characteristic that is applicable to all brain cancers. Indeed, brain tumor cells share the characteristic of being iron-dependent, and ferritin is a key player in iron transport across the BBB [113], indicating that high ferritin in patients with brain tumors would be expected. Obtaining more comprehensive data on serum ferritin levels in patients with multiple brain cancer types is essential to accurately establish its contributing role in brain cancers.

### Ferritin in brain cancer cells

Elevated ferritin complex levels are observed for intracellular iron storage in brain cancer cells. Specifically, the FTL subunit is upregulated in GBM-derived cells [114], as well as in GBM stem-like cells [3], where both FTH and FTL levels correlate with glioma grade and disease aggressiveness. In addition, in a cohort of 171 patients with tumors ranging from grade I to IV, FTL and FTH expression levels were found to increase with tumor grade and high levels were associated with a significantly worse prognosis [115]. However, another cohort study containing astrocytomas and GBM showed that high FTL, but not FTH levels, correlated with shorter survival times [116]. GBM patients in this cohort showed no difference in survival between high and low expression of FTH and FTL [116]. Further studies with larger datasets are thus required to draw more definitive conclusions on the link between ferritin expression and brain tumor aggressiveness. Furthermore, studies investigating the iron dependency of brain CSCs highlight a growing interest in the biological role of ferritin in these cells, especially given its potential involvement in maintaining their stem-like properties and promoting therapeutic resistance. A close correlation between intracellular ferritin expression and cancer cell stem identity has been established in other cancers, such as breast cancer [117, 118]. Similarly, the FTL subunit is preferentially imported into breast CSC-enriched spheres compared to their differentiated counterparts [102]. Moreover, the use of a synthetic derivative of salinomycin (AM5) in breast CSCs induced iron depletion both in vitro and in vivo, mechanistically via the degradation of ferritin within lysosomes, leading to increased ROS production and subsequent selective breast CSCs death via ferroptosis [101].

Concerning brain cancers, it has been reported that GBM stem-like cells import significantly higher amounts of iron by upregulating TF and its receptor TFR1 [3]. Expression of TFR1 also correlated with a higher frequency of the GBM tumorosphere formation, increased tumor incidence, and decreased survival of mice carrying GBM tumors with high TFR1 expression [3]. Both in vitro and in vivo studies showed that knockdown of either the H or L subunits of ferritin in GBM CSCs markedly reduced cellular growth, as well as their capacity to form tumors in orthotopic models, which supports the role of ferritin as a key player in the proliferation and invasion of CSCs [3]. In the context of CSC self-renewal and tumorigenicity in GBM, molecular analysis revealed a strong correlation between ferritin content, STAT3, and the transcription factor FoxM1 [119]. Furthermore, Wu and colleagues reported that FTL levels are significantly higher in patients with GBM in comparison with those with lower-grade glioma [114]. Similar to the effect on GBM CSCs observed by Schonberg and colleagues, significant impairment of GBM cell growth upon FTL knockdown was achieved [114]. The mechanism underlying this inhibition was attributed to the activation of the GADD45A/JNK pathway coupled to a substantial decrease in Wnt target genes (Cyclin D1;c-Myc). Finally, in vitro delivery of FTH1 siRNA in patient xenograft-derived CSCs from GBM and suppression of FTH1 (and thus decreased iron availability) resulted in reduced CSC viability, most likely due to overwhelming oxidative stress in these cells [120].

While the studies reviewed above highlight the importance of both iron import and storage to strongly advocate for targeting ferritin as a strategy to eliminate CSCs, a few studies advise caution in pursuing this approach. Namely, targeting ferritin in ovarian and breast CSCs has been shown to increase cancer aggressiveness and promote cell proliferation [121, 122]. These observations suggest that the cellular consequences of ferritin modulation may vary significantly depending on the tumor type and its cellular origin. An important, but often overlooked, aspect of iron metabolism in cancer is the functional utilization of iron, beyond its intracellular concentration. Mechanistically, total intracellular iron alone is not the whole story—how a tumor uses iron (for DNA repair, energy metabolism, redox buffering, etc.) and the cell’s lineage-specific biology determine whether ferritin acts mainly as a protective iron reservoir or as a regulator of metabolism and redox state [3, 88]. We therefore propose a simple, testable framework: CSCs from embryonal/embryonic-like tumors may depend on ferritin chiefly to buffer iron and preserve stemness under fluctuating microenvironments, whereas CSCs from differentiated epithelial tumors may exploit iron in specialized metabolic or redox processes, In this context, ferritin loss can trigger stress-adaptive programs (proliferation, migration, EMT) that paradoxically increase aggressiveness [123–125]. This model reconciles conflicting observations and highlights the need for tumor-type-specific evaluation of ferritin-targeting strategies. Furthermore, an additional level of complexity arises when considering the incorporation of extracellular ferritin by tumor cells. Interestingly, it has been shown that extracellular ferritin uptake can reduce the invasion of the GBM CSCs [126]. Although this might come as a surprise, the authors of this study argue that high uptake of ferritin can be seen as a proxy for a nutrient-rich environment. According to the “go or grow” hypothesis (invade/proliferate dichotomy), cancer cells tend to increase their invading capacity when nutrient availability is limiting, while nutrient-rich environments correlate with increased proliferation [127, 128].

However, targeting ferritin in epithelial tumors should not be completely ruled out as removal of the iron buffering system within the cell makes them more susceptible to iron-dependent (ferroptotic) cell death [121, 122]. Unfortunately, no measurements of free iron in these FTH-silenced CSCs were performed, but it is plausible that increased levels of free iron would be expected. Combined, these findings highlight the critical role of iron and ferritin in regulating the proliferation and invasion of CSCs in brain tumors, demonstrating that, depending on the availability of the LIP, CSCs modulate their cellular fate. However, the lack of comprehensive studies exploring the precise mechanisms underlying this modulation leaves significant gaps in our understanding and warrants further investigation.

## Targeting ferritin as a new therapeutic approach

**Fig. 4:**
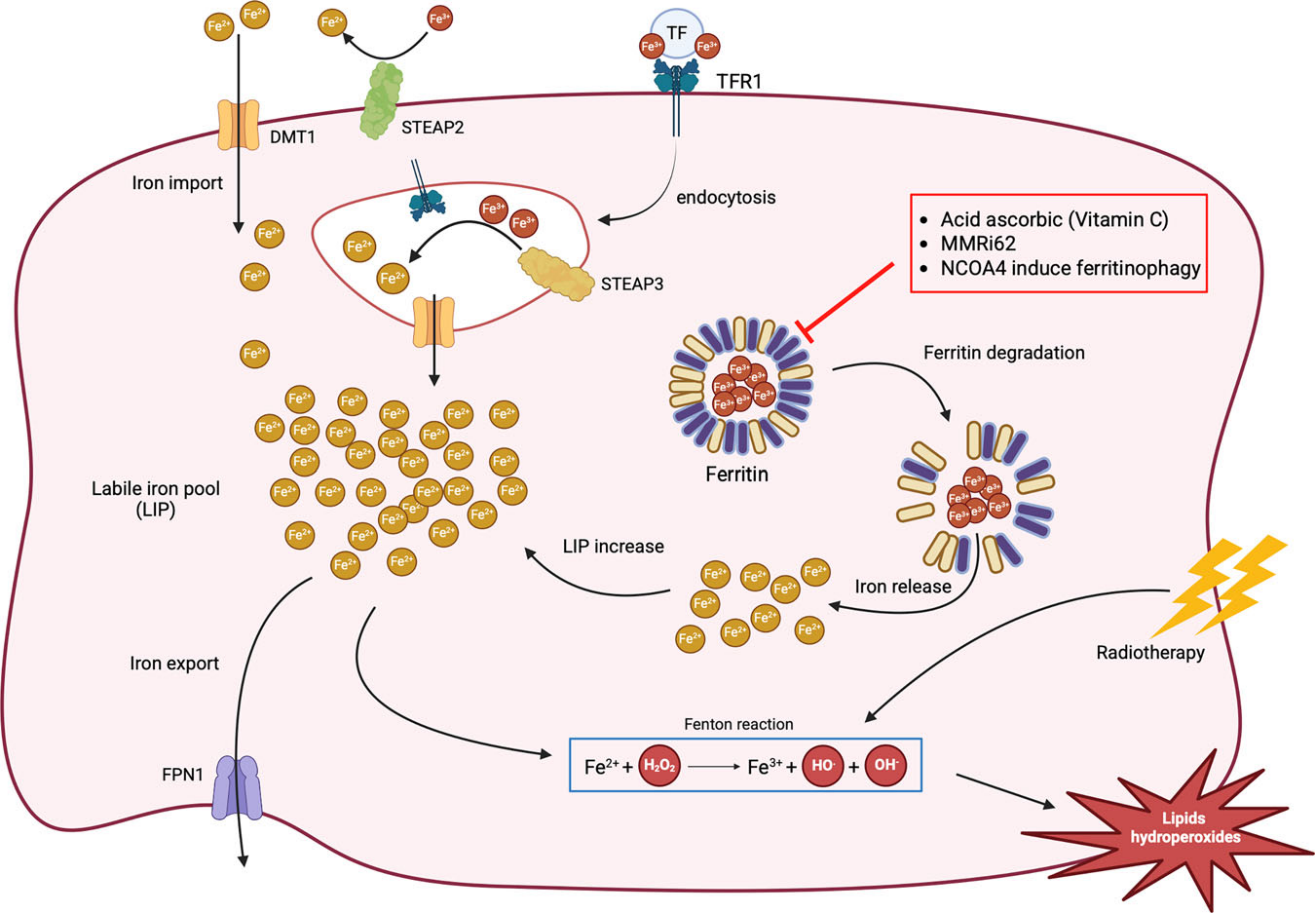
Cancer cell sensitization by ferritin targeting. Representative schematic of ferroptosis induction in cancer cells via a systemic increase in intracellular iron levels, mediated by ferritin degradation through NCOA4 activation or treatment with MMRi62/Vitamin C. Consequently, free ferrous iron (Fe²⁺) reacts with ROS, promoting lipid hydroperoxide formation and triggering ferroptotic cell death. This effect can be further enhanced by radiotherapy, which increases ROS production and can be used as a complementary treatment. DMT1 divalent metal transporter 1, FPN1 ferroportin-1, H_2_O_2_ hydrogen peroxide, HO. hydroxide ion, MMRi62 7-[(2,3-dichlorophenyl)-(pyridin-2-ylamino) methyl]quinolin-8-ol, NCOA4 nuclear receptor coactivator 4. OH− hydroxyl radical, ROS reactive oxygen species, STEAP2 six-transmembrane epithelial antigen of prostate 2, STEAP3 six-transmembrane epithelial antigen of prostate 3, TF transferrin, TFR1 transferrin receptor 1. *Created with BioRender.com*.

Currently, therapeutic attempts to decrease iron *via* iron chelation have not proven to be clinically efficient for cancer patients. Furthermore, these studies lack specificity and do not target the CSCs that provide resistance to conventional therapies. Here, we propose that a more effective strategy moving forward would be to remove the ferritin buffering capacity of iron to achieve tumor-specific targeting of iron-dependent cell death, such as ferroptosis. Indeed, as previously highlighted, cancer cells and CSCs harbor high requirements for iron. Consequently, these cells are potentially susceptible to iron toxicity if ferrous iron (Fe²⁺) levels were to be elevated rapidly and react with ROS via the Fenton reaction to cause ferroptosis. Therefore, the buffering of cellular iron via ferritin forms a central vulnerability point for these metabolically ‘iron addicted’ cells. For the last decade, ferroptosis induction has presented a powerful new strategy for the eradication of cancer cells (as reviewed in ref. [129]). Considering the important role that ferritin plays as an antioxidant complex (Fig. 1), this targeted reduction of ferritin in brain cancers has the potential to either induce direct cell death (potentially via ferroptosis) or to increase sensitivity to conventional therapies that act to increase ROS levels, and thus, iron sensitivity (Fig. 4).

### Ferritinophagy induction as therapeutical approach

The induction of ferritinophagy has emerged as a cellular mechanism of interest to sensitize both cancer stem and non-stem cells to ferroptosis [130]. The principle behind this strategy is intended to cause a sudden release of stored iron from ferritin within cancer cells that would fuel the Fenton reaction and lead to ferroptosis (Fig. 4). Previously, inhibition of ferritinophagy by targeting the NCOA4-FTH1 interaction was shown to reduce the LIP, thereby inhibiting ferroptosis and promoting cell survival [131]. Inhibiting NCOA4-mediated ferritinophagy limits iron release and Fenton chemistry, and consequently, oxidative stress, highlighting the close relationship between ferritinophagy and ferroptosis induction. Conversely, it has been shown that increased NCOA4 by knockdown of COPZ1 (coatomer protein complex subunit zeta 1) results in the degradation of ferritin and induction of ferroptosis in GBM [132]. In addition, ubiquination of NCOA4 by TRIM7 using K48-linked chains has been shown to reduce ferritinophagy and facilitate growth and therapeutic resistance of GBM. This study suggests that by targeting TRIM7, which is overexpressed in GBM, we can increase NCOA4 activity and sensitize cancer cells to ferroptosis [133]. Ferritinophagy can also be induced by cystine deprivation that ultimately leads to ferroptosis [134], a mechanism that we have studied extensively in numerous cancer cell types via manipulation of cystine transport mechanisms [135, 136]. Recently, ferritinophagy-inducing molecules have shown encouraging pre-clinical effects in cancer models. MDM2-MDM4 RING inhibitors (MMRi) such as MMRi62 and MMRi71, which were initially designed to inhibit the MDM2–MDM4 interaction to stabilize p53 [137], have recently been demonstrated to trigger the death of cancer cells. Specifically, MMRi62 promoted ferroptosis in pancreatic cancer cells by accelerating lysosomal degradation of the ferritin complex subunit FTH and increasing the iron availability within the cell [138] (Fig. 4). In leukemia, MMRi71 has been demonstrated to promote ferritin degradation, leading subsequently to the induction of apoptosis [139]. Regarding the use of MMRi for brain tumors, further studies are needed to evaluate its efficacy in crossing the BBB and the specificity of targeting cancer cells, as well as to assess off-target neurotoxicity.

### Ferritin removal by vitamin C

In recent years, a renewed interest in high-dose vitamin C as an anti-cancer therapy has been investigated, demonstrating its efficacy against a wide variety of cancers, including the brain [140, 141]. Mechanistically, it is noteworthy that 1 mM of vitamin C can activate ferritinophagy, leading to ferritin degradation (Fig. 4) and a form of cell death exhibiting ferroptosis-like features in thyroid cancer cells [142]. Additionally, our data revealed that high doses of vitamin C induce rapid ferritin degradation in MB cell lines, resulting in abrupt increases in cellular iron bioavailability (unpublished data). At these concentrations, vitamin C causes prompt cell death, which is prevented by iron chelation, but not by ferrostatin-1, a specific ferroptosis inhibitor. These results suggest that vitamin C-induced cell death is iron-dependent but cannot be qualified as ferroptosis. Further investigations are required to elucidate the precise mechanism by which vitamin C activates ferritinophagy. Nevertheless, this pathway represents a promising avenue for targeting ferritin in cancer therapy.

### Enhancing radiotherapy/chemotherapy efficacy through ferritin targeting

Brain cancers exhibit a strong resistance to conventional treatments such as radiotherapy and chemotherapy. Therefore, identifying targets that can sensitize cancer cells to these treatments while minimizing long-term side effects is essential (Fig. 4). Brain cancer patients are particularly vulnerable to cognitive impairments, which is especially true for pediatric cancers like MB, which predominantly affect children under the age of 16 and frequently experience severe, long-term intellectual deficits following treatment [143].

Our laboratory and others have established a close link between radiotherapy and ferroptosis. Ionizing radiation causes DNA damage that leads to cell-cycle arrest or cell death, and also generates ROS through water radiolysis, promoting lipid peroxidation and ferroptosis [144, 145]. Radiotherapy increases ACSL4 levels, thereby enhancing PUFA synthesis and expanding the pool of oxidizable lipids [146]. Furthermore, radiotherapy depletes GSH, thereby impairing the GSH/GPX4 antioxidant defense pathway, which renders cells more susceptible to ferroptosis [147]. In MB cell lines, low doses of radiation induce ferroptosis. Moreover, reducing GPX4 expression by inhibiting mTORC1 activity through integrin αVβ3 targeting enhances the effect of radiation on these cells [145].

Radiotherapy resistance represents one of the major challenges in oncology and the presence of CSCs seems to play a pivotal role in this regard [148]. One hypothesis for this resistance is that by increasing ferritin-mediated iron storage, cells reduce the pool of free iron and thereby limit the formation of hydroxyl radicals (HO^•^). Considering the high expression of ferritin in brain CSCs [3], it is highly likely that CSCs also utilize ferritin as a factor contributing to radiotherapy resistance. In line with this is the report of an increased number of lipid droplets correlating with elevated levels of FTH in radioresistant lung cancer cells [118].

The hypothesis that ferritin contributes to radiotherapy resistance may also be pertinent in the context of chemotherapeutic resistance. Cancer cells resistant to chemotherapy frequently exhibit dysregulated iron metabolism. Temozolomide (TMZ), the standard first-line chemotherapeutic agent for glioma, encounters therapeutic resistance in approximately 60% of patients. Emerging evidence suggests that alterations in iron metabolism and differential sensitivity to ferroptosis are key mechanisms underlying GBM progression and treatment resistance [149, 150]. In a recent GBM cohort study, increased expression of FTH and FTL chains was found to correlate with elevated GPx4 levels, a key regulator of ferroptosis, thereby contributing to both ferroptosis resistance and TMZ resistance. Liu and colleagues employed cationic liposomes to deliver siRNA targeting ferritin in various tumor models, including glioma and breast cancer. Silencing ferritin expression via this method significantly enhanced the sensitivity of tumor cells to the DNA-alkylating agent carmustine (BCNU), a chemotherapeutic commonly used in glioma treatment [151]. Corroborating these findings, both FTH and FTL overexpression have also been implicated in chemotherapy resistance across various tumor types and chemotherapeutic agents (reviewed in ref. [152].

## Clinical challenges and perspective

Targeting ferritin degradation and ferroptosis in brain cancers represents a promising therapeutic strategy due to their dependence on iron metabolism. A variety of compounds have demonstrated the ability to induce ferritinophagy, increase labile iron, and trigger ROS-mediated ferroptotic cell death in preclinical models (detailed in Table 1). However, translating these findings into clinical application faces several critical challenges. Foremost, effective delivery to the CNS is limited by the BBB, which restricts systemic drug penetration. Many of these agents, including peptide-based and hydrophobic small molecules, exhibit poor CNS bioavailability, necessitating advanced delivery systems such as nanoparticles, convection-enhanced delivery, or BBB permeabilizing strategies [113, 153]. Additionally, the intrinsic mechanisms of action, primarily ROS generation and labile iron accumulation, pose a significant risk of neurotoxicity. Off-target ferroptosis in neurons and glial cells could result in irreversible neurological damage, highlighting the need for tumor-specific targeting and controlled dosing regimens [154, 155]. Pharmacokinetic limitations, such as rapid metabolism and short half-lives, further complicate achieving therapeutic concentrations within brain tumors while minimizing systemic toxicity.

**Table 1:** Pharmacological profiles and translational challenges of ferritin-targeting agents in brain tumors.

Compound	Mechanism of action	Direct/indirect ferritin degradation	Study subject	Reference	Challenges for clinical translation (brain-focused)
Salinomycin/ironomycin (AM5)	Induces ferritinophagy via lysosomal iron accumulation; sequesters iron, triggers autophagy-mediated ferritin degradation and ferroptosis.	Indirect	Breast cancer stem cells	[101]	Limited BBB penetration; potential neurotoxicity; need for tumor-specific delivery
DeFer-2	PROTAC molecule that recruits ferritin to the proteasome for ubiquitin-mediated degradation; decreases ferritin and increases labile iron pool.	Direct	Murine melanoma cells	[158]	Poor CNS bioavailability, stability and off-target effects; delivery across the BBB
Itaconate	Activates NCOA4-mediated ferritinophagy; promotes ferritin degradation and iron release, enhancing ferroptosis	Indirect	Mouse models, cell lines	[159]	BBB permeability; risk of neurotoxicity due to ROS; controlled dosing challenges
JQ1	Inhibits BRD4, leading to transcriptional activation of autophagy genes; promotes NCOA4-mediated ferritinophagy and ferroptosis	Indirect	Cell lines	[160]	CNS penetration; specificity for tumor vs. healthy cells; pharmacokinetic limitations
DpdtC	Iron chelation-independent ferritinophagy inducer; mobilizes intracellular iron, increases ROS via Fenton reaction, sensitizing cells to ferroptosis	Indirect	Gastric carcinoma cells	[161]	Potential neurotoxicity from ROS; delivery challenges across the BBB; systemic side effects
MMRi62	Lysosomal degradation of ferritin via NCOA4-dependent autophagy; increases labile iron and ROS, triggering ferroptotic cell death	Direct	Pancreatic cancer cells	[138]	BBB crossing; off-target effects in brain cells; balancing efficacy and toxicity
Caryophyllene oxide	Induces ferritinophagy through NCOA4 recruitment and lysosomal degradation; increases intracellular iron and ROS, promoting ferroptosis	Indirect	Liver cancer cells	[162]	BBB permeability; ROS-mediated neurotoxicity; targeted delivery needed
Astragalin	Activates NCOA4-mediated ferritinophagy in fibrotic liver tissue; enhances labile iron and ferroptosis of fibrotic cells	Indirect	Hepatic stellate cell lines	[163]	CNS delivery unknown; uncertain ferroptosis induction in brain tumors
Dihydroartemisinin (DAT)	Ferritin degradation via lysosomal sequestration of iron; ROS accumulation triggers ferroptosis in an autophagy-independent manner	Indirect	Leukemia cell lines	[164]	BBB permeability limited; ROS-related neurotoxicity risk; systemic toxicity concerns
Vitamin C	Reduces ferritin via ROS-mediated ferritinophagy; inactivates GPX4, increases labile iron, and promotes lipid peroxidation leading to ferroptosis	Direct	Anaplastic thyroid cancer cells	[142, 165]	Poor BBB crossing at therapeutic doses; need for targeted delivery
Ce6-PEG-HKN15	Targets ferritin using HKN15 peptide conjugate; upon laser activation, it induces ROS-mediated ferritin degradation	Direct	Cancer cell lines	[166]	Delivery to CNS tumors; requirement of laser activation limits application; neurotoxicity risk

Several clinical trials have investigated high-dose vitamin C as a potential modulator of iron metabolism in cancer therapy. However, these studies were not specifically designed to evaluate ferritin modulation or the relationship between vitamin C treatment and ferritin response in patients [156, 157]. Despite these challenges, the unique vulnerability of brain tumor cells to iron-dependent oxidative stress provides a compelling rationale for continued development. Future clinical strategies may involve combination therapies that sensitize tumor cells to ferritinophagy while sparing healthy CNS tissue, or the use of BBB-penetrant delivery vehicles. Moreover, integrating predictive biomarkers of iron metabolism and ferroptotic susceptibility could refine patient selection and optimize therapeutic efficacy. In conclusion, while clinical translation is constrained by delivery, toxicity, and pharmacokinetic hurdles, ferritin-targeted approaches offer a novel and mechanistically rational avenue for treating aggressive brain cancers, warranting further preclinical optimization and early-phase clinical investigation.

## Conclusion

Iron is an indispensable micronutrient for metabolism, yet its redox activity is potentially harmful. Cancer cells often exhibit elevated iron requirements to sustain proliferation, while tumor-initiating cancer stem cells (CSCs) display an even greater dependency, reflected by increased ferritin expression and iron uptake. Ferritin, as a central regulator of intracellular iron, has therefore emerged as a potential therapeutic vulnerability across various malignancies.

Ferritin-targeted strategies remain largely underexplored in brain cancers and could represent a novel approach to induce ferroptosis in embryonic-origin tumors, such as glioblastoma (GBM) and medulloblastoma (MB), potentially sensitizing CSCs to conventional therapies by reducing their antioxidant capacity.

Emerging approaches, such as modulating ferritin turnover or altering labile iron pools, illustrate the therapeutic potential of targeting iron storage. However, these strategies remain highly experimental, and substantial efforts are required to clarify their mechanisms, safety, and specificity. Critical challenges include achieving effective blood–brain barrier penetration, minimizing neurotoxicity and off-target ferroptosis, addressing tumor heterogeneity in iron metabolism, and understanding compensatory pathways that regulate iron homeostasis.

In summary, ferritin stands out as a compelling and multifaceted target at the crossroads of iron metabolism, redox regulation, and cancer stem cell survival. Its central role in buffering intracellular iron and protecting cells from oxidative stress positions it as a potential vulnerability in aggressive brain tumors, such as GBM and MB. However, translating this potential into effective therapies requires a much deeper understanding of ferritin biology. Rigorous studies are needed to elucidate the precise mechanisms by which ferritin modulates ferroptosis and CSC resistance. By addressing these knowledge gaps, future research can pave the way for the rational development of ferritin-targeted clinical strategies to enhance therapeutic response, overcome resistance, and improve outcomes in brain cancer patients.
